# Enhancing microcirculation in STEMI patients: can intracoronary thrombolysis combined with thrombus aspiration provide an optimal strategy?

**DOI:** 10.3389/fcvm.2025.1516054

**Published:** 2025-03-28

**Authors:** DongDong Yan, WenQiang Li, Ming Bai, Pei Wang, Zheng Zhang

**Affiliations:** ^1^ Department of Cardiology, First Hospital of Lanzhou University, Lanzhou, China; ^2^ First Clinical Medical College of Lanzhou University, Lanzhou, China

**Keywords:** ST-elevation myocardial infarction, intracoronary thrombolysis, thrombus aspiration, MVO, optimal strategy

## Abstract

ST-elevation myocardial infarction (STEMI) is a critical cardiovascular emergency characterized by acute coronary artery occlusion and subsequent myocardial injury. The current standard of care is primary percutaneous coronary intervention (PPCI), which aims to rapidly restore epicardial blood flow. However, despite successful revascularization, microvascular obstruction (MVO) remains a major challenge, contributing to adverse clinical outcomes. This article explores the potential role of intracoronary thrombolysis, in conjunction with thrombus aspiration, in improving microcirculatory perfusion during PCI for STEMI patients. The pathophysiology of MVO is systematically reviewed, followed by an evaluation of clinical studies on thrombus aspiration and intracoronary thrombolysis in STEMI management. Furthermore, the potential benefits of combining these two approaches in mitigating MVO are discussed. Finally, the clinical evidence is critically assessed, existing controversies are analyzed, and directions for future research are proposed.

## Introduction

ST-elevation myocardial infarction (STEMI) is a severe manifestation of coronary artery disease, characterized by the sudden occlusion of a coronary artery, leading to significant myocardial injury. The primary objective in STEMI management is the rapid restoration of coronary blood flow, most commonly achieved through percutaneous coronary intervention (PCI) (1–3). However, despite successful reperfusion, microvascular obstruction (MVO) may still occur, resulting in suboptimal myocardial perfusion and unfavorable clinical outcomes (4).

Microvascular obstruction (MVO) is a critical complication following myocardial ischemia and subsequent reperfusion in STEMI. It is characterized by the obstruction of small coronary microvessels, leading to impaired myocardial perfusion. This phenomenon is driven by multiple interrelated mechanisms, including direct thrombotic obstruction, inflammatory responses, and the accumulation of toxic metabolites (5). Studies have demonstrated that MVO is associated with adverse clinical outcomes, such as larger infarct size and impaired left ventricular function. Patients with MVO following STEMI are at a higher risk of developing complications, including heart failure, arrhythmias, and increased mortality (6). Understanding the pathophysiology of MVO is essential for developing effective therapeutic strategies to improve microvascular perfusion and enhance clinical outcomes in STEMI patients. Despite PCI's efficacy in restoring epicardial blood flow, MVO remains a significant challenge that can negatively impact patient prognosis (7). Current clinical approaches aim to mitigate MVO through pharmacological interventions and mechanical strategies, such as thrombus aspiration. While certain pharmacological trials have shown that early intervention within a specific time window may reduce MVO extent following reperfusion therapy, most clinical studies have yielded unsatisfactory results. Therefore, further research is required to identify optimal therapeutic agents or strategies for the prevention and treatment of MVO.

Thrombus aspiration is a mechanical technique used to remove thrombotic material from the coronary artery during or prior to PCI. Its theoretical benefits stem from the potential to enhance myocardial perfusion by reducing the residual thrombus burden within the coronary arteries. However, clinical trial outcomes have been inconsistent. While earlier studies suggested that thrombus aspiration could be beneficial in the treatment of STEMI, more recent trials have failed to demonstrate a clear advantage, leading to ongoing debate regarding its clinical utility.

Intracoronary thrombolysis is another approach for reducing intracoronary thrombotic burden in patients with STEMI. With the advent of the PCI era and the widespread adoption of coronary interventional procedures, intracoronary thrombolysis has been largely replaced by coronary stent implantation. However, while PCI effectively addresses epicardial coronary artery lesions, it remains ineffective in treating MVO. Studies indicate that nearly half of patients experience MVO following PCI. Unlike PCI alone, intracoronary thrombolysis has the potential to target both epicardial coronary thrombi and microvascular obstruction. Evidence suggests that intracoronary thrombolysis can improve Thrombolysis in Myocardial Infarction (TIMI) flow and Myocardial Blush Grade (MBG) in STEMI patients. Despite these theoretical advantages, recent clinical trial results have been largely disappointing. Notably, studies using major adverse cardiovascular events (MACE) as the primary endpoint have failed to demonstrate significant benefits (8). This may be attributed to the substantial reduction in STEMI-related mortality achieved through PCI alone, potentially diminishing the relative impact of adjunctive therapies. Given these findings, the combination of intracoronary thrombolysis with thrombus aspiration may offer a complementary strategy to enhance therapeutic outcomes in STEMI patients.

**Figure 1:**
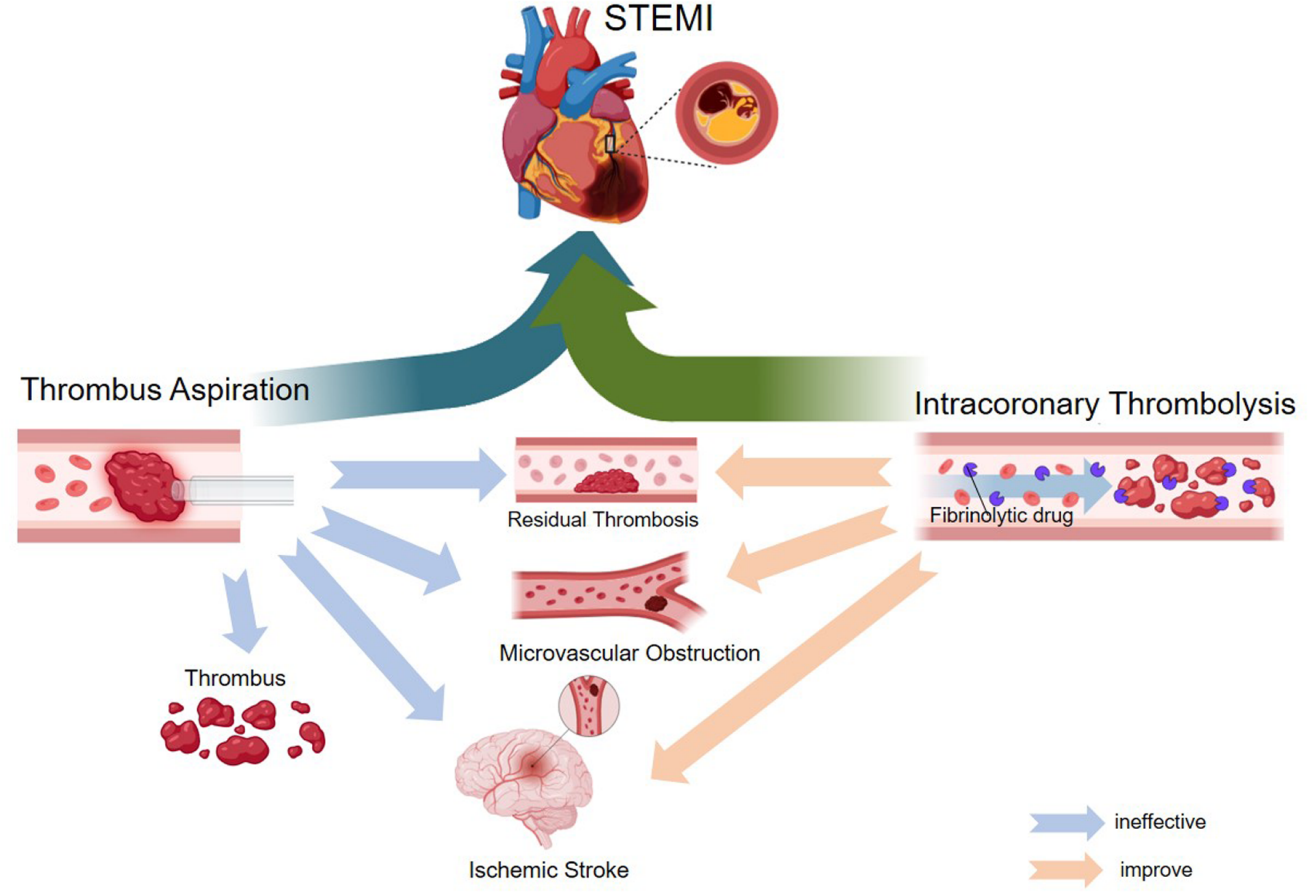
Mechanism diagram of the application of intracoronary thrombolysis as an adjunct therapy for thrombus aspiration in STEMI.

The routine use of intracoronary thrombolysis and thrombus aspiration in STEMI treatment presents several challenges and ongoing controversies. While the theoretical benefits of these interventions are well-established, inconsistencies in clinical trial outcomes have led to uncertainty regarding their overall efficacy. A key issue is the identification of patient subgroups that may derive the greatest benefit, as the lack of universally accepted guidelines complicates clinical decision-making. As research on MVO continues to evolve, the potential role of intracoronary thrombolysis and thrombus aspiration in improving microvascular perfusion in STEMI patients remains a crucial area of investigation (Figure 1).

### Pathophysiology of MVO in STEMI

MVO is a critical complication that arises following myocardial ischemia and subsequent reperfusion in STEMI. It is characterized by the obstruction of small coronary microvessels, leading to impaired myocardial perfusion. MVO is closely associated with total ischemia time and infarct size in STEMI patients—the longer the duration of ischemia, the more severe the degree of MVO. This phenomenon is driven by multiple interrelated mechanisms. First, the presence of thrombus within the coronary vasculature can directly occlude microvessels, leading to ischemic injury in the surrounding myocardial tissue. Thrombus formation occurs rapidly following the rupture of an atherosclerotic plaque, triggering platelet aggregation and the release of pro-coagulant factors that exacerbate clot development. This process not only obstructs larger coronary arteries but also extends into the microcirculation, resulting in significant perfusion deficits (9). Second, inflammatory responses triggered by myocardial injury can lead to endothelial dysfunction and vasoconstriction, further exacerbating MVO. Ischemia activates inflammatory pathways, promoting the release of cytokines and other inflammatory mediators that compromise microvascular integrity (10). This inflammatory cascade increases endothelial permeability, leading to the accumulation of plasma proteins and fluid in the interstitial space, contributing to edema and MVO (11). In particular, polymorphonuclear neutrophils and their formation of neutrophil extracellular traps (NETs) have drawn increasing attention in the context of coronary atherothrombosis (12). Third, the accumulation of toxic metabolites during ischemia and reperfusion further contributes to microvascular damage. Upon reperfusion, oxidative stress and the generation of reactive oxygen species (ROS) can induce endothelial cell injury and apoptosis, setting off a cascade of events that ultimately leads to MVO (10). Additionally, metabolic byproducts such as lactate can accumulate during prolonged ischemia, exacerbating tissue damage and perpetuating microvascular dysfunction (13).

In addition to the intrinsic pathophysiological mechanisms of MVO caused by STEMI itself, interventional procedures in STEMI patients can also influence its occurrence. Despite the effectiveness of PCI in restoring coronary blood flow, MVO remains a significant challenge that can adversely impact patient outcomes. Current clinical strategies aim to mitigate MVO through various interventions, including pharmacological agents and mechanical approaches such as thrombus aspiration. The introduction of drug-eluting stents and adjunctive pharmacological therapies, such as antiplatelet agents, has significantly improved clinical outcomes in STEMI patients. However, even after successful PCI, MVO may still develop, underscoring the need for additional interventions to optimize microvascular perfusion (1). Studies have shown that interventional procedures can promote distal embolization due to the dislodgement of thrombotic material, potentially reducing the overall benefits of revascularization (14). Additionally, repeated post-dilation following stent implantation has been associated with slow or absent coronary flow. Although the precise mechanisms remain unclear, the clinical consequences can be severe (15). Research indicates that post-dilation during PPCI is linked to poor prognosis (16), as it predisposes patients to vasospasm, endothelial injury, and platelet activation, thereby exacerbating reperfusion injury. These factors are considered primary contributors to slow or no-reflow phenomena (17). Furthermore, STEMI patients exhibit a hypercoagulable state, which may be further aggravated by the introduction of interventional devices into the bloodstream (18).

Studies have demonstrated that MVO is associated with adverse clinical outcomes, including larger infarct size and impaired left ventricular function (19). Patients who develop MVO following STEMI are at a higher risk of complications such as heart failure, arrhythmias, and increased mortality (20). Understanding the pathophysiology of MVO is essential for developing therapeutic strategies aimed at improving microvascular perfusion and enhancing overall prognosis in STEMI patients. Current guidelines recommend several pharmacological interventions for the prevention and treatment of MVO, including intracoronary (IC) administration of high-dose adenosine or sodium nitroprusside (SNP), among others (21). However, due to significant individual variability among STEMI patients and differences in interventional approaches, clinical management cannot rely on a single drug or method to effectively mitigate MVO. Instead, a multimodal strategy combining pharmacological agents and procedural techniques is commonly employed in clinical practice to minimize MVO severity and improve outcomes.

### Thrombus aspiration in STEMI

Thrombus aspiration involves the mechanical removal of thrombotic material from the coronary artery during or prior to PCI. This technique aims to enhance coronary blood flow and reduce the risk of MVO by physically extracting obstructive thrombi. Specialized aspiration catheters are designed to capture and remove thrombus while minimizing damage to the surrounding vasculature (22). Despite its theoretical benefits, the routine clinical use of thrombus aspiration remains controversial (23). The TAPAS trial was the first clinical study to assess whether thrombus aspiration could improve prognosis in STEMI patients. The results suggested that, compared with conventional PCI, thrombus aspiration before stent placement in the infarct-related artery was associated with improved one-year clinical outcomes following PCI (24). However, subsequent large-scale trials yielded conflicting results. The TASTE trial, which evaluated the clinical benefit of routine thrombus aspiration in STEMI, found no reduction in 30-day mortality (25). Similarly, the later TOTAL trial demonstrated that routine thrombus aspiration did not provide additional clinical benefit (26). Consequently, guidelines from the American College of Cardiology/American Heart Association (ACC/AHA) recommend thrombus aspiration only in select patient populations, acknowledging the mixed evidence regarding its efficacy (3).

More recent studies suggest that thrombus aspiration may be particularly beneficial in patients with a large thrombus burden or high-risk features (27, 23). However, the lack of consensus on the optimal timing and patient selection complicates clinical decision-making, underscoring the need for continuous updates based on emerging research. Additionally, growing evidence suggests that adjunctive therapies, such as glycoprotein IIb/IIIa inhibitors or novel antiplatelet agents, may enhance the efficacy of thrombus aspiration (28). These agents act synergistically to reduce platelet aggregation and facilitate thrombus resolution, potentially improving outcomes in patients undergoing PCI with thrombus aspiration. However, the optimal timing, choice of pharmacological agents, and overall treatment strategy require careful consideration to maximize therapeutic benefits while minimizing risks (29).

With the publication of the TAPAS, TASTE, and TOTAL trial results, evidence has increasingly suggested that thrombus aspiration does not provide additional clinical benefit in the management of STEMI (Table 1). Consequently, several clinical guidelines have downgraded the recommendation for thrombus aspiration in STEMI patients (30). However, despite these guideline revisions, thrombus aspiration remains widely utilized in clinical practice. This discrepancy raises a fundamental question: does the issue lie within the guidelines themselves, or does it stem from variations in clinical practice? In current clinical settings, the appropriate selection of patients who may derive the greatest benefit from thrombus aspiration and other adjunctive therapies is crucial. Risk assessment tools and advanced imaging modalities can aid in identifying patients with a high thrombus burden or an increased risk of MVO (26). Incorporating these tools into clinical decision-making may enable more precise patient selection, thereby optimizing the benefits of thrombus aspiration and improving overall outcomes in STEMI management.

**Table 1:** List of recent clinical RCTs of thrombus aspiration during PPCI in patients with STEMI.

Study	Size	Ischaemic type and time	Intervention group	Comparator group	Main findings
Tone Svilaas et al*.* (31) TAPAS-trial	*n* = 1,071	STEMI < 12 h	thrombus-aspiration group + PCI	conventional-PCI group	The primary end point was a MBG of 0 or 1 and the thrombus-aspiration group is better.
Ole Fröbert et al*.* (25) TASTE-trial	*n* = 7,244	STEMI < 24 h	Manual thrombus- aspiration + PCI	PCI only	No difference in the primary end point was all-cause mortality at 30 days
SanjitS Jolly et al*.* (32) TOTAL-trial	*n* = 10,732	STEMI < 12 h	Manual thrombus- aspiration + PCI	PCI alone	No difference in the primary outcome was a composite of death from cardiovascular causes, recurrent myocardial infarction, cardiogenic shock, or New York Heart Association (NYHA) class IV heart failure within 180 days.

### Intracoronary thrombolysis in STEMI

Total coronary occlusion is observed in approximately 87% of patients within the first four hours after symptom onset; however, this proportion decreases significantly when patients are evaluated 12–24 h after symptom onset (33). The use of intracoronary plasminogen activators and thrombolytic therapy in STEMI patients has been documented in clinical practice for decades (34) Since the rapid advancement of coronary interventions in the 1980s, numerous research centers have conducted clinical studies investigating the efficacy of intracoronary thrombolysis for STEMI.

For instance, Anderson et al. (35) conducted a study in which 50 patients with acute myocardial infarction were randomly assigned to receive either intracoronary streptokinase or standard therapy within approximately three hours of symptom onset. The study found that streptokinase administration alleviated pain, as evidenced by reduced morphine use. Additionally, patients treated with streptokinase demonstrated significant improvement in Killip classification (*P* < 0.01), and echocardiographic wall-motion index analysis revealed greater functional recovery compared to the control group (*P* < 0.01). Similarly, Rentrop et al. (36) compared the effects of intracoronary streptokinase and intracoronary nitroglycerin infusion on coronary angiographic patterns and mortality in patients with acute myocardial infarction. The study reported that acute recanalization occurred in 74% (32 of 43) of patients receiving streptokinase, whereas only 6% (1 of 18) of patients treated with nitroglycerin alone achieved recanalization. Interestingly, in 1985, Kennedy et al. (37) conducted a randomized trial evaluating the long-term outcomes of intracoronary streptokinase in acute myocardial infarction, reporting the results of a 12-month follow-up. A total of 134 AMI patients were randomized to receive intracoronary streptokinase, while 116 control patients received standard care. Although initial differences between the two groups were not statistically significant, a significant survival benefit emerged during the first year (*P* = 0.03).

With the advent of the stent era, the widespread adoption of stent implantation has significantly reduced mortality and reinfarction rates in STEMI patients. Consequently, clinical management has increasingly prioritized mechanical revascularization and stent implantation as the primary treatment strategies. While stent therapy offers substantial benefits, no single intervention is without limitations. Notably, stent implantation primarily addresses stenosis and obstruction in the epicardial coronary arteries but does not directly influence the coronary microcirculation. As a result, studies have shown that MVO remains prevalent in STEMI patients even after successful stent implantation. In recent years, growing awareness of this issue has prompted researchers to explore pharmacological and interventional strategies aimed at improving coronary microcirculation, with intracoronary thrombolytic therapy emerging as a potential approach.

Theoretically, intracoronary thrombolysis facilitates thrombus dissolution, improves TIMI blood flow, and enhances myocardial perfusion—key therapeutic goals in STEMI management. Effective myocardial perfusion is not only critical for reducing MVO but also for improving long-term patient prognosis. Consequently, numerous research institutions have conducted clinical trials investigating the impact of intracoronary thrombolysis on MVO in STEMI patients. Several large-scale studies have yielded noteworthy findings (Table 2). Murat Sezer et al. (38) evaluated the effect of intracoronary streptokinase (ICSK) administered immediately after primary PCI (PPCI) on long-term left ventricular infarct size, volumes, and function. Ninety-five patients undergoing PPCI were randomized to receive ICSK 250 kU (*n* = 51) or no additional therapy (*n* = 44). At 6-month follow-up, the ICSK group exhibited significantly smaller infarct size and lower left ventricular end-systolic and end-diastolic volumes. Additionally, ejection fraction was significantly higher in the ICSK group compared with the control group (57.2% vs. 51.8%; *p* = 0.018). McCartney et al. (39) conducted a randomized clinical trial evaluating the effect of low-dose intracoronary alteplase administered during PPCI on MVO in patients with acute myocardial infarction. Wang et al. (40) found that patients in the treatment group had a significantly higher incidence of complete ST-segment resolution (STR) at 90 min post-PPCI and a greater proportion of myocardial blush grade (MBG) 3 compared to the control group. Huang et al*.* (8) reported that adjunctive intracoronary pro-urokinase or tirofiban administered before stent implantation during PPCI improved myocardial reperfusion without increasing the incidence of major bleeding events. But Jiang et al*.* (41) found no statistically significant difference in major adverse cardiovascular events (MACE) between the treatment and control groups at the 6-month follow-up (*P* > 0.05).

**Table 2:** List of recent clinical RCTs of intracoronary thrombolysis to improve MVO in patients with STEMI.

Study	Size	Ischaemic type and time	Intervention group	Comparator group	Main findings
Murat Sezer et al*.* (38)	*n* = 95	STEMI < 12 h	Streptokinase 250 kU	Standard care	At 6 months, infarct size and left ventricular end-systolic and end-diastolic volumes were significantly smaller, and the ejection fraction was significantly higher in the ICSK group compared with the control group
McCartney et al*.* (39)	*n* = 440	STEMI < 12 h	Alteplase 10 mg or 20 mg	Volume matched saline (20 ml)	No difference with alteplase vs. placebo for the primary endpoint, i.e., MVO assessed by CMR (at 2–7 d).
Wang et al. (40)	*n* = 182	STEMI < 12 h	Pro-urokinase 20 mg	Volume matched saline (10 ml)	Patients in study group had a higher incidence of complete STR and MBG 3 compared with those in control group.
Huang et al*.* (8)	*n* = 354	STEMI < 12 h	Pro-urokinase 20 mg or Tirofiban (10 μg/kg)	Volume matched saline (20 ml)	Final CTFC after PCI was significantly lower in the pro-urokinase (*P* < 0.001) and tirofiban (*P* < 0.001) groups than in the saline group and similar between the pro-urokinase and tirofiban groups (*P* > 0.05).
Jiang et al*.* (41)	*n* = 260	STEMI < 12 h	Pro-urokinase10 mg	Volume matched saline (10 ml)	At 6-months follow-up, there was no statistically different of MACE between the two groups (*P* > 0.05).

### Intracoronary thrombolysis as an adjunct to thrombus aspiration

The theoretical benefits of intracoronary thrombolysis and thrombus aspiration lie in their potential to enhance myocardial perfusion by addressing residual thrombus burden within the coronary arteries. Intracoronary thrombolysis, when used as an adjunct to thrombus aspiration, may augment thrombus resolution while simultaneously reducing the risk of embolic events caused by thrombus detachment, potentially yielding a synergistic effect greater than the sum of its individual components. Both interventions aim to improve coronary perfusion, as reflected by higher TIMI flow grades and improved myocardial blush grade (MBG), which serve as indicators of effective reperfusion in ischemic myocardial tissue (42). Emerging evidence suggests that the combined application of these techniques may lead to improved clinical outcomes, particularly in patients with a high thrombus burden (43). Some studies have reported that early intracoronary thrombolysis may reduce the risk of MVO and facilitate myocardial recovery, especially when performed in conjunction with PCI (44). By promoting thrombus dissolution and facilitating the rapid restoration of blood flow, intracoronary thrombolysis may help mitigate myocardial injury and preserve left ventricular function. Additionally, this combined approach has been associated with reductions in infarct size, lower incidence of heart failure, and improved survival rates in high-risk patient populations (45). However, despite these promising theoretical advantages, the clinical efficacy of intracoronary thrombolysis and thrombus aspiration remains a subject of debate. While some studies have demonstrated potential benefits, large-scale randomized trials have yielded conflicting results, underscoring the need for further research to delineate the optimal patient selection criteria, procedural strategies, and long-term impact of these interventions.

Recent studies have aimed to elucidate the efficacy of intracoronary thrombolysis and thrombus aspiration in improving clinical outcomes in STEMI patients. A systematic review of randomized controlled trials demonstrated that while intracoronary thrombolysis significantly enhanced myocardial perfusion parameters, its impact on long-term outcomes remained inconsistent (46). The heterogeneity in study designs, patient selection criteria, and procedural protocols poses a challenge in drawing definitive conclusions regarding its role in contemporary STEMI management. Similarly, the clinical benefits of thrombus aspiration remain a topic of debate. While some studies suggest that thrombus aspiration may confer advantages in select populations—particularly those with a high thrombus burden—its overall efficacy has been questioned due to conflicting trial results (47).

Although both thrombolysis and thrombus aspiration appear to yield immediate improvements in myocardial perfusion, their influence on long-term outcomes, including survival and functional recovery, remains uncertain. Notably, some studies indicate that short-term improvements in perfusion do not necessarily correlate with sustained clinical benefit or long-term survival (48). This discrepancy underscores the need for further investigations into the long-term implications of these interventions, particularly their impact on patient quality of life and functional recovery. As emerging evidence continues to refine the understanding of these strategies, future treatment guidelines and clinical protocols will need to integrate the latest findings. Given the variability in trial methodologies and patient characteristics, a cautious and individualized approach is warranted when interpreting these results and applying them to clinical practice (46).

### Clinical controversies and challenges

The routine application of intracoronary thrombolysis and thrombus aspiration in the management of STEMI remains a subject of considerable debate. While the theoretical benefits of these interventions are well-established, discrepancies in clinical trial outcomes have led to uncertainty regarding their overall efficacy. For instance, although some studies suggest that thrombus aspiration may mitigate microvascular obstruction (MVO), others—such as the TASTE trial—have failed to demonstrate a significant improvement in long-term clinical outcomes (49). Moreover, these procedures are associated with inherent risks, including distal embolization, an increased incidence of stroke, and potential complications related to thrombolytic therapy. In particular, distal embolization poses a major concern, as it may exacerbate microvascular dysfunction and offset the potential benefits of thrombus removal (27). The challenge of patient selection further complicates clinical decision-making, as there are currently no universally accepted guidelines to identify individuals who may derive the greatest benefit from these interventions (50). Consequently, clinicians must carefully weigh the potential advantages of these strategies against their associated risks and uncertainties, emphasizing an individualized approach to optimize patient outcomes.

Current clinical guidelines advocate for a cautious approach to the use of thrombus aspiration and intracoronary thrombolysis, emphasizing individualized treatment strategies tailored to patient-specific characteristics and clinical presentation (1). The heterogeneity in trial outcomes has underscored the need for more stringent patient selection criteria and standardized procedural protocols to optimize therapeutic efficacy. Consequently, ongoing discussions within the cardiology community focus on refining evidence-based recommendations to assist clinicians in making informed decisions regarding the appropriate use of these interventions. Establishing clearer guidelines is essential to ensuring consistency in clinical practice and improving patient outcomes.

### Future directions

With advancements in STEMI management and treatment strategies, the mortality rate of STEMI patients has declined to a certain extent, yet further reductions remain challenging. In recent years, extensive research has been conducted to explore the pathophysiological mechanisms and clinical management of MVO. Among potential therapeutic strategies, intracoronary thrombolysis, when used as an adjunct to thrombus aspiration, has emerged as a promising approach for the prevention and treatment of MVO. Future research should focus on several key areas:

• Personalized Treatment Approaches

Developing personalized treatment strategies based on individual patient characteristics—such as thrombus burden and microvascular integrity—may enhance the efficacy of thrombus aspiration and intracoronary thrombolysis. The integration of advanced imaging modalities, such as optical coherence tomography (OCT) and intravascular ultrasound (IVUS), can provide detailed assessments of thrombus composition and microvascular status. These technologies enable more precise, tailored interventions, potentially maximizing therapeutic benefits while minimizing procedural risks.

• Technological Innovations

Continued advancements in catheter and thrombectomy device technology are essential to enhancing the safety and efficacy of thrombus aspiration. The development of novel devices aimed at minimizing distal embolization and improving thrombus retrieval may lead to better clinical outcomes for patients undergoing PCI. Ongoing innovations in catheter design and aspiration techniques will be crucial for overcoming the limitations of current approaches and optimizing procedural success.

• Further Clinical Trials

Large-scale, well-designed clinical trials are essential to comprehensively assess the long-term impact of these interventions on clinical outcomes. Future studies should extend beyond mortality and myocardial infarction rates to evaluate functional recovery, quality of life, and cost-effectiveness (51). Additionally, research focusing on the specific indications for thrombus aspiration and thrombolysis in diverse patient populations will provide a more refined understanding of their role in STEMI management.

• Exploring Alternative Therapies

Exploring complementary therapies, including novel antiplatelet agents and vasodilators, may further enhance the efficacy of thrombus aspiration and thrombolysis (52). Additionally, a deeper understanding of the underlying mechanisms of MVO could identify new therapeutic targets, complementing existing interventions and opening new avenues for improving patient outcomes (53).

• Training and Standardization

Enhancing training for interventional cardiologists on the indications, techniques, and potential complications of thrombus aspiration and thrombolysis is crucial. Establishing standardized protocols will ensure consistent and safe application across diverse clinical settings.

## Conclusion

Intracoronary thrombolysis, as an adjunct to thrombus aspiration, presents a promising strategy for improving microvascular perfusion in STEMI patients. Despite inconsistencies in research findings and ongoing clinical controversies, it remains widely utilized in the prevention and treatment of MVO. As the understanding of MVO mechanisms deepens, the efficacy of this combination therapy is expected to be further validated.
